# P-867. Management of Positive Urine Cultures in Kidney Transplant Recipients Within Sixty Days Post Transplantation

**DOI:** 10.1093/ofid/ofaf695.1075

**Published:** 2026-01-11

**Authors:** Sumeet Jain, Patricia Saunders-Hao, Kirby An, Nicholas Jandovitz, Esther Benamu, Edisson Ospina-Sanchez

**Affiliations:** North Shore University Hospital, Westbury, NY; North Shore University Hospital, Westbury, NY; North Shore University Hospital, Westbury, NY; North Shore University Hospital, Westbury, NY; Transplant Institute, Northwell Health, Manhasset, New York; North Shore University Hospital - Northwell Health, Manhasset, New York

## Abstract

**Background:**

Asymptomatic bacteriuria (ASB) frequently occurs in kidney transplant recipients (KTRs) and is often treated with antibiotics despite unclear benefits. Current guidelines from the American Society of Transplantation recommend a five-day antibiotic regimen for ASB within the first two months post-transplant (PT), although this recommendation is not supported by robust evidence.

**Figure:**
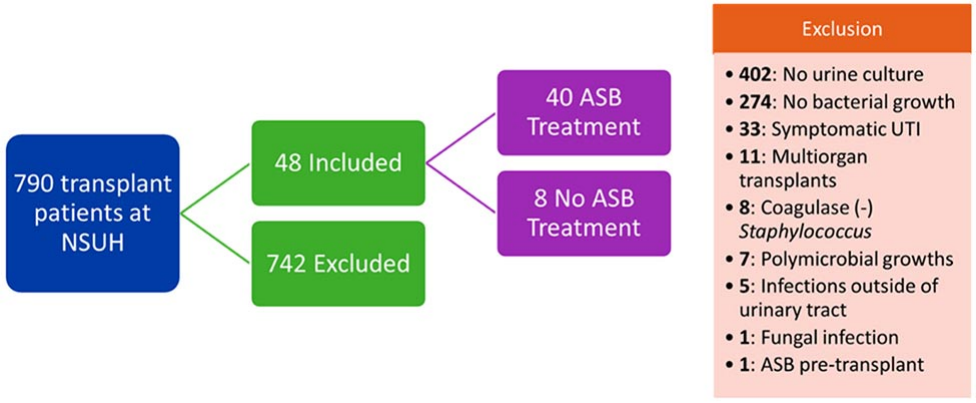
Flow chart of patient screening for eligibility

**Figure:**
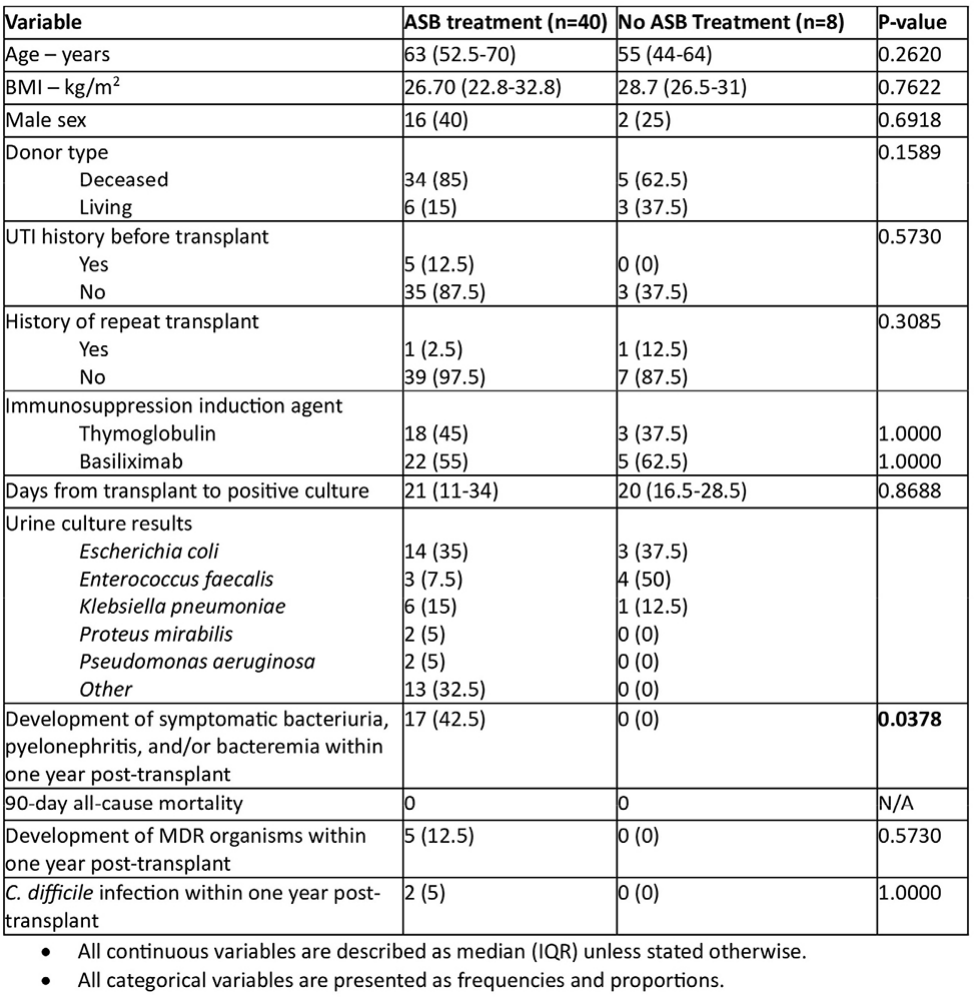
Comparison of baseline characteristics and treatment outcomes between treatment and no treatment groups

**Methods:**

This IRB-exempt, retrospective chart review evaluated adult KTRs with a positive urine culture within the first 60 days PT between January 2016 to September 2023. Patients were included if they had a positive urine culture without documentation of urinary symptoms. Patients were excluded if they had polymicrobial urine cultures, fungal urinary infections, multiorgan transplant recipients or receipt of antibiotics for indications other than prophylaxis. The primary outcome compared the incidence of symptomatic bacteriuria, pyelonephritis and/or bacteremia from a urinary source within one-year PT between patients who received therapy and those who did not. Secondary outcomes included 90-day all-cause mortality, development of multi-drug resistant (MDR) bacteria, adverse events such as *Clostridioides difficile* infection (CDI) and comparison of durations of therapy.

**Figure:**
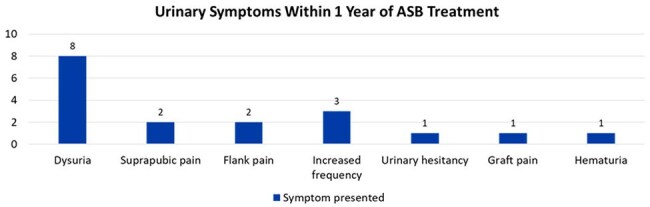
Urinary symptoms in patients who developed symptomatic bacteriuria Symptoms reported by patients in those who developed symptomatic bacteriuria, including pyelonephritis and bacteremia from urinary source (symptoms not exclusive, patients may have reported one or more symptoms)

**Results:**

A total of 790 KTRs were screened, of which 48 were included in final analysis with 40 in the treatment group and 8 in the no treatment group. Symptomatic bacteriuria occurred in 17 patients (42.5%) in the treatment group versus 0 in the no treatment group (p = 0.0378). Nearly 90% of treated patients received ≥ 5 days of antibiotics. Five patients (12.5%) in the treatment group developed MDR organisms within one year versus 0 patients in the no treatment group. CDI occurred in 2 patients (5%) in the treatment group versus 0 patients in the no treatment group.

**Conclusion:**

This study showed that providing ASB treatment in the 60 days PT was associated with increased incidence of subsequent symptomatic bacteriuria, though it was underpowered to demonstrate statistical significance. There was also a trend toward higher rates of MDR organisms and CDI in treated patients. These findings suggest the recommendation to treat ASB in KTRs early PT may need reconsideration, though larger studies are needed to confirm our findings.

**Disclosures:**

All Authors: No reported disclosures

